# Excellent Thermoelectric Performance Realized in Copper Sulfide Magnetic Nanocomposites Via Modified Solid States Reaction

**DOI:** 10.1002/advs.202409494

**Published:** 2025-02-08

**Authors:** Tian‐Yu Yang, Zi‐Yuan Wang, Xi Yan, Chong‐Yu Wang, Yi‐Xin Zhang, Zhen‐Hua Ge, Jing Feng

**Affiliations:** ^1^ Faculty of Materials Science and Engineering Kunming University of Science and Technology Kunming 650093 China

**Keywords:** copper sulfide, Cu_1.8_S, Fe_3_O_4_, ferromagnetic oxide, thermoelectric performance

## Abstract

Solid‐state reactions, which are the basic reactions involved in the preparation, processing, and application of materials, are ubiquitous in material science and chemistry research. In the fields of metallurgy and geology, a significant number of complex chemical reactions occur during the smelting of ores. Inspired by the smelting of copper concentrate, this work applies modified metallurgical chemical reactions to the field of (thermoeletric) TE materials. By controlling the reaction temperature and composition, porous copper sulfide magnetic nanocomposites can be formed. Regulating the composition generates numerous precipitates and Cu‐rich phases, and controlling the microstructure facilitates the formation of porous structures. The second phase and porous structure effectively decreased thermal conductivity. Furthermore, the introduction of ferromagnetic Fe_3_O_4_ particles plays a role in reducing carrier concentration and forming potential barrier scattering low energy carriers, which improves the Seebeck coefficient of the samples. Ultimately, the optimum figure of merit (ZT) of ≈1.3 at 773 K for the Cu_1.8_S + 2 wt.% Fe_3_O_4_ bulk sample and an average ZT of 0.57 over the entire operating temperature range. The modified solid states reaction between oxides and sulfides could be employed to optimize electrical and thermal transport properties for sulfide TE material, as well as sulfide batteries, sulfide photoelectric materials, and sulfide catalytic materials.

## Graphical abstract

Inspired by the smelting of ores, this work applies modified metallurgical chemical reactions to the field of (thermoelectric) TE materials. The results show that the average power factor is increased by 26% and the thermal conductivity is reduced by 70% over the entire operating temperature range. Finally, the optimal sample obtaines a figure of merit (ZT) value of 1.3 at 773 K, an increase of 180%. The average ZT is 0.57 in the temperature range of 323–773 K.

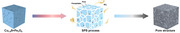

## 1 Introduction

The new epoch has been developed since the late 20th century because of technological progress in semiconductors, such as transistors, diodes, solar cells, piezoelectric transducers, and TE materials. However, advances in technology are inseparable from the basic chemical reactions. It is precisely because of the study of the ubiquitous chemical reaction that scientific research can be further developed by revealing the mechanism behind the reaction. TE materials enable the conversion of heat to electricity and vice versa, thus facilitating new energy generation and solid‐state cooling.^[^ ^1^ ^]^ The theoretical sources of TE materials are the Seebeck and Peltier effects,^[^ ^2^ ^]^ the optimization process of TE performance is also accompanied by many chemical reactions. Based on this theory, the dimensionless figure of merit (ZT) is used to evaluate the TE performance of materials, namely, ZT = S^2^σT/κ, where S is the Seebeck coefficient, σ is the electrical conductivity, T is the absolute temperature, and κ is the thermal conductivity. The thermal conductivity (κ) is composed of the electronic thermal conductivity (κ_e_) and lattice thermal conductivity (κ_l_). The most straightforward strategy for maximizing the TE performance is either enhancing the power factor (PF = S^2^σ) or reducing the thermal conductivity (κ = κ_e_ + κ_l_), or both at the same time.

Copper sulfide (or selenide) is a new liquid‐like TE material that possesses properties consistent with the phonon‐liquid electron‐crystal (PLEC) concept.^[^ ^3^ ^]^ The mobile superionic Cu ions are kinetically disordered in the rigid sublattice formed by sulfur atoms. This trait provides a new strategy for the design of low‐thermal conductivity materials. Numerous studies have been conducted on optimizing TE performance. Common strategies include stoichiometric ratio regulation,^[^ ^4^ ^]^ element doping,^[^ ^5^ ^]^ entropy engineering,^[^ ^6^ ^]^ compositing,^[^ ^7^ ^]^ ion blocking,^[^ ^8^ ^]^ band engineering,^[^ ^9^ ^]^ nanoprecipitation,^[^ ^10^ ^]^ lattice engineering,^[^ ^11^ ^]^ and boundary engineering.^[^ ^12^ ^]^ The main response of these methods is to optimize the electrical conductivity or to decrease the thermal conductivity. The limitations of the TE performance of Cu_1.8_S are mainly attributed to its low Seebeck coefficient and high thermal conductivity. The primary research objective is to effectively suppress thermal conductivity and simultaneously enhance the Seebeck coefficient by regulating the carrier concentration.

In the field of metallurgy, 82% of copper is smelted via pyrometallurgy, which is accompanied by numerous chemical reactions.^[^ ^13^ ^]^ The chalcocite (Cu_2_S) is the main mineralogical species in the concentrate, and the regular chemical reaction during pyrometallurgy is as follows:^[^ ^14^ ^]^

(1) $$Cu_{2}S\left(s\right)+2CuO\left(s\right)\overset{\Delta }{\to }4Cu\left(s\right)+SO_{2}\left(g\right)$$

In this reaction, the element S was oxidized, the valance of S changed from −2 to +4, and the element Cu was reduced from +1 and +2 to 0. The Cu_2_S is the oxidizing agent and also the reducing agent. The smelting temperature range of the above reaction is above 1100–1350 °C. A similar reaction occurs between Bi_2_S_3_ (bismuthinite) and Bi_2_O_3_, as shown below:^[^ ^15^ ^]^

(2) $$2Bi_{2}S_{3}\left(s\right)+Bi_{2}O_{3}\left(s\right)\overset{\Delta }{\to }Bi\left(l\right)+SO_{2}\left(g\right)$$

The two regular solid states reactions inspired the new strategy to enhance the thermoelectric properties of metal sulfides: 1) the addition of the oxides could modify the concentration of metal ions in the matrix for optimizing the carrier concentration; 2) the SO_2_ gas could introducing the pores for reducing the lattice thermal conductivity.

Here, we designed a modified solid states reaction for improving the thermoelectric and mechanical properties of Cu_1.8_S‐based materials with not only the two advantages mentioned above but also element doping and second phases.

**Figure 1:**
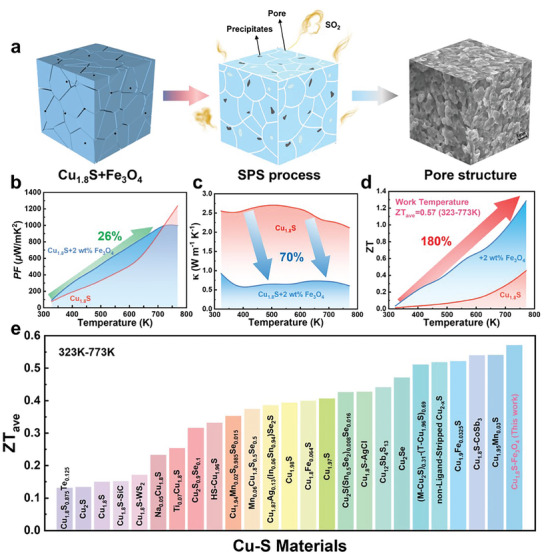
a) Schematic of the formation of copper sulfide magnetic nanocomposites. After Fe_3_O_4_ was added to Cu_1.8_S, SO_2_ was formed via the SPS process, and a porous structure was formed. b) The difference in power factor between the pure Cu_1.8_S and optimum component–doped samples. The average power factor of the doped sample was 26% higher than that of the undoped sample. c) The difference in thermal conductivity between the pure Cu_1.8_S and optimum component–doped samples. The average thermal conductivity of the doped sample was 70% that of the undoped sample. d) The difference in ZT between the pure Cu_1.8_S and optimum component–doped samples. The average ZT of the doped sample was 180% higher than that of the undoped sample. e) Average ZT values of the Cu─S materials in the temperature range 323–773 K, as reported in the literature.^[^ ^4^, ^6^, ^7^, ^8^, ^16^ ^]^

**Figure 2:**
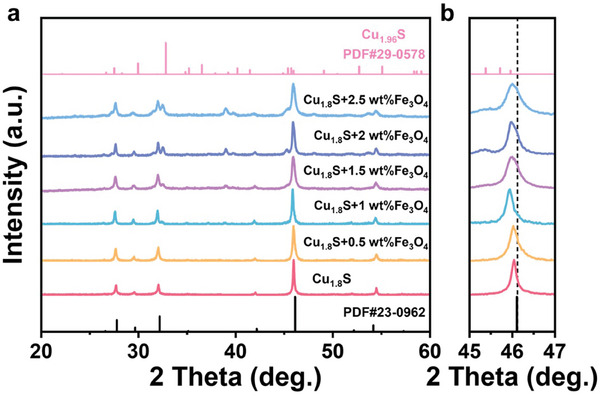
Phase structures of the Cu_1.8_S + x wt.% Fe_3_O_4_ (x = 0, 0.5, 1, 1.5, 2, 2.5) bulk samples. a) X‐ray diffraction (XRD) pattern of the Fe_3_O_4_‐doped samples. b) Magnified XRD pattern for 2 Theta values between 45° and 47°.

The magnetic Fe_3_O_4_ particles were added to the Cu_1.8_S matrix according to a stoichiometric ratio of x wt.% Fe_3_O_4_ (x = 0, 0.5, 1, 1.5, 2, 2.5) and sintered by spark plasma sintering to form a magnetic nanocomposite, as shown in **Figure**  **1**. The addition of Fe_3_O_4_ causes a series of in situ solid‐state reactions during the sintering process to control the composition and microstructure. The reaction parameters (such as temperature and composition) are very important for the redox reaction and directly determine the thermoelectric properties. In the field of metallurgy, the smelting temperature range of the above reaction is above 1100–1350 °C. However, in this work, the sintering temperature is fixed at 500 °C because 500 °C is an optimized temperature for sintering Cu_1.8_S powder to bulk. The controlled reaction is not conducted completely due to the low reaction temperature, which is why there are still Fe_3_O_4_ particles in the sintered bulk. However, the incomplete reaction is good for formatting the complex microstructure of the sintered samples and benefits from the high thermoelectric properties. The additional contents of Fe_3_O_4_ in Cu_1.8_S have been well investigated and discussed in the manuscript. With the increase of Fe_3_O_4_, the content of pores is increased, and the Fe doping content in the matrix is also increased. However, there is a limitation of Fe in the Cu_1.8_S lattice, it is therefore the reaction is finished when the Fe_3_O_4_ contents are beyond 1.5 wt.% (**Figure**  **2**), and the excess Fe_3_O_4_ remains in the matrix as a dispersed second phase. The second phase (Cu_1.96_S and Fe_3_O_4_) and porous structure effectively decreased the thermal properties and ferromagnetic particles Fe_3_O_4_ increased the Seebeck coefficient. The related reaction equation can be roughly given as follows:

(3) $$Cu_{1.8}S\left(s\right)+xFe_{3}O_{4}\left(s\right)\overset{\Delta }{\to }Cu_{1.8}Fe_{x}S_{1-2x}\left(s\right)+2xSO_{2}\left(g\right)$$

According to the smelting reaction of ore, the addition of oxides will generate SO_2_. Therefore, the addition of Fe_3_O_4_ to copper sulfide can not only release SO_2_ to form the porous structure but also realize Fe doping. The carrier concentration was modified by Fe doping and the generating of S vacancies, resulting in the 26% enhancement of the average power faction (Figure  1b). The introduction of Fe_3_O_4_ particles affects the electrical transport properties of non‐magnetic Cu_1.8_S to a certain extent. The reason is that the magnetic particles scatter some carriers and affect the carrier mobility and electron conductivity. Due to the generating of SO_2_, the pores remained in the Cu_1.8_S. The kinetics of redox reactions do not change during the SPS process because the content of reactant Fe_3_O_4_ is very low compared to the Cu_1.8_S. The remaining Fe_3_O_4_ in the Cu_1.8_S matrix is due to the lower sintering temperature and the limitation of Fe in the matrix, which is beneficial to improve the thermoelectric properties. The second phases and pores benefit from reducing the lattice thermal conductivity (70% decrement, as shown in Figure  1c). As a result, the ZT value of the Cu_1.8_S + 2 wt.% Fe_3_O_4_ sample gets a 180% increment compared to that of the pristine sample, reaching 1.3 at 773 K. It is worth mentioning that the average ZT from (323 to 773 K) is 0.57 of the magnetic nanocomposites, this is an ultra‐high value in copper sulfides based thermoelectric materials (as shown in Figure  1e). This work demonstrated that the addition of oxide particles is beneficial to modifying the composition and introducing pores and second phases in metal sulfides. Our strategy could be also utilized to enhance TE properties for other sulfide materials, such as Bi_2_S_3_, Ag_2_S, PbS, Cu_3_SbS_4_, and Cu_2_ZnSnS_4_.

## 2 Results and Discussion

### 2.1 Phase Structure

The phase structures of the Cu_1.8_S + x wt.% Fe_3_O_4_ (x = 0, 0.5, 1, 1.5, 2, 2.5) bulk samples were characterized by X‐ray diffraction (XRD) (Figure  2). The peak of the pure sample corresponded to the hexagonal Cu_1.8_S phase (PDF#23‐0962), i.e., the room temperature (RT) phase. With the addition of Fe_3_O_4_, the sample diffraction peak began to detect some Cu_1.96_S (PDF#29‐0578), which we attributed to the volatilization of sulfur.^[^ ^17^ ^]^ The previous study demonstrated that S volatilization can generate an S vacancy, and the presence of an S vacancy induces the collapse of the lattice frame.^[^ ^18^ ^]^ The rejection of cations in anion vacancies to the greatest extent possible is a distinctive phenomenon in sulfides; an anion vacancy induces lattice expansion.^[^ ^19^ ^]^ The evolution of the diffraction peak matches this phenomenon well. With the increase of Fe_3_O_4_, the diffraction peaks gradually shift toward a low angle. When the addition contents to 1.5 wt.%, the solid solution limit of samples is reached and the diffraction peaks no longer shift. Besides, when the additional contents are above 1.5 wt.%, there is an obvious diffraction peak of Cu_1.96_S appearing in the XRD patterns. Subsequently, the 2 wt.% doped sample was analyzed using the Rietveld refinement method, and the lattice constant of all samples is shown in Figure S8 (Supporting Information).

### 2.2 Thermoelectric Performance

**Figure 3:**
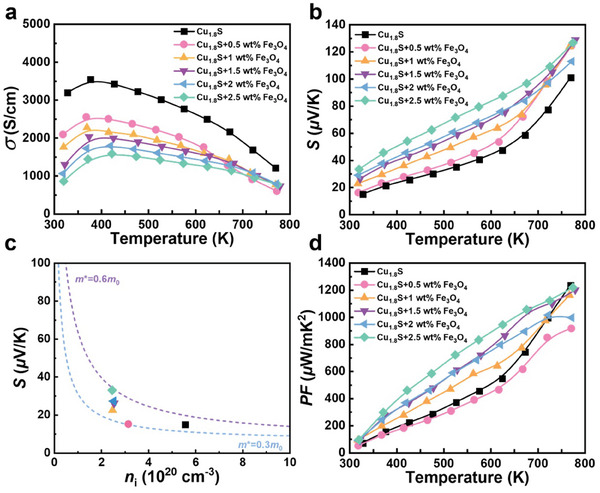
Electric transport performance of the Cu_1.8_S + x wt.% Fe_3_O_4_ (x = 0, 0.5, 1, 1.5, 2, 2.5) bulk samples. Temperature dependence of a) electric conductivity, b) Seebeck coefficient, and d) power factor. c) Seebeck coefficient as a function of carrier concentration at 300 K, as fitted using Pisarenko curves based on the single parabolic band model.

The temperature dependence in the TE performance of the Cu_1.8_S + x wt.% Fe_3_O_4_ (x = 0, 0.5, 1, 1.5, 2, 2.5) bulk samples are shown in **Figure**  **3**. The electrical conductivity (σ) initially increased with temperature and then decreased, demonstrating that the semiconductor behavior changed to degenerate semiconductor behavior. The pure sample exhibited ultrahigh electrical conductivity, whereas the Fe_3_O_4_‐doped sample exhibited decreased electrical conductivity at all temperatures after the addition of Fe_3_O_4_. In this study, Fe_3_O_4_ was added to the matrix, because it acts as a p‐type dopant and the carrier scattering center, which reduces the carrier concentration and mobility (Figure S7, Supporting Information), so the electrical conductivity decreased slightly. The inflection point of the electrical conductivity represents the phase transition (PT) temperature point in the copper sulfides, and the PT temperature of the Cu‐rich phase (namely, Cu_1.96_S and Cu_2_S) shifts toward higher temperatures. This phenomenon corresponds to the XRD results. With the addition of Fe_3_O_4_, Cu_1.96_S was formed. When the Fe_3_O_4_ content reached a certain extent, the electrical conductivity no longer deteriorated. The Seebeck coefficient (S) is shown in Figure  3b. Positive Seebeck coefficients for all samples demonstrated p‐type conductive behavior, with the main carrier being the hole. The Seebeck coefficient increased with the addition of Fe_3_O_4_, which can be attributed to the role of the interfaces scattering some low‐energy carriers and the increased density of states. The discussion of interface barrier scattering carriers is detailed in the magnetic properties part, and the increased density of states is detailed in the Supporting Information. To further determine the effects of the nanoprecipitate phase on the carrier transport properties of copper sulfides, Hall tests were performed at 323 K. Figure S7 (Supporting Information) shows the carrier concentration (n) and mobility (μ) of the Cu_1.8_S + x wt.% Fe_3_O_4_ (x = 0, 0.5, 1, 1.5, 2, 2.5) bulk samples at RT. The ultrahigh electrical conductivity of pure Cu_1.8_S can be attributed to a higher hole concentration. The value of n decreased from 5.56 × 1020 cm^−3^ for the pure sample to 2.49 × 1020 cm^−3^ for the Cu_1.8_S + 2 wt.% Fe_3_O_4_ sample. With the addition of Fe_3_O_4_, the value of μ gradually decreased from 33.4 to 18 cm^2^ V^−1^ s^−1^ for the Cu_1.8_S + 2 wt.% Fe_3_O_4_ sample. Owing to the Seebeck coefficient being optimized effectively over the entire temperature range, the average power factor was 26% higher than that of the pure Cu_1.8_S sample.

**Figure 4:**
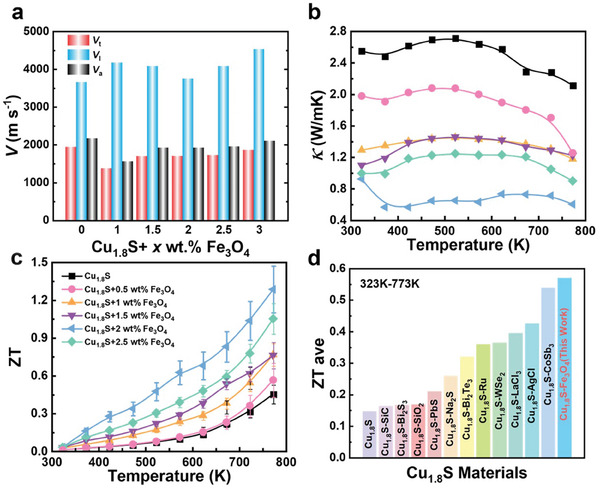
Thermal transport properties and TE performance of the Cu_1.8_S + *x* wt.% Fe_3_O_4_ (*x* = 0, 0.5, 1, 1.5, 2, 2.5) bulk samples. a) Sound velocity of all samples, temperature dependence of b) thermal conductivity and c) ZT, and d) comparison of the average ZT for Cu_1.8_S materials in the temperature range 323–773 K.

The sound velocities of all samples were measured by the ultrasonic reflection method. The results are presented in **Figure**  **4**a. The longitudinal sound speed (vl) initially decreased and then increased after adding Fe_3_O_4_, with the 2 wt.% doped samples exhibiting the lowest vl. The phonon transmission speed indicates the introduction of interfaces and phase boundaries, which effectively reduce the sound velocity. In addition, the transverse sound speed (vt) and average sound speed (va) were maintained at comparable levels. The related elastic properties and calculation formulas of the parameters are presented in Table S1 (Supporting Information). The previous study proved that porous interfaces are more stable than nanoprecipitates at high temperatures and strengthen phonon scattering.^[^ ^11d^ ^]^ The presence of porous structure effectively enhanced phonon scattering to optimize thermal conductivity in Cu_1.8_S + 2 wt.% Fe_3_O_4_. After doping 2 wt.% Fe_3_O_4_ in Cu_1.8_S, iron oxide nanoprecipitates were observed in the matrix. Different from the smelting of copper concentrate (>1100 °C), this is because the reaction is not complete due to insufficient temperature. Therefore, the continuous addition of Fe_3_O_4_ will lead to more iron oxide left in the matrix. It is well known that the thermal conductivity of oxides is high.^[^ ^20^ ^]^ Based on the Debye–Callaway model, $\tau_{B}^{-1}=\frac{v}{d}$, where τB, v, and d are the phonon relaxation time, sound velocity, and average grain size, respectively. The larger grain size corresponds to a longer phonon relaxation time, which weakens phonon scattering and increases thermal conductivity.^[^ ^21^ ^]^ Unfortunately, there is no accurate way to detect carrier conductivity in Cu_1.8_S material systems because of the existence of ion conductivity and because we failed to obtain the lattice thermal conductivity. Because of the influence of various defects on phonon transport, the total thermal conductivity of Cu_1.8_S + 2 wt.% Fe_3_O_4_ was lower than that of all samples over the entire temperature range, and the thermal conductivity was reduced to 0.58 W m^−1^ K^−1^ at 773 K. Thereafter, the dimensionless figure of merit ZT was optimized over the entire temperature range after adding Fe_3_O_4_. Ultimately, the optimal ZT value of the Cu_1.8_S + 2 wt.% Fe_3_O_4_ sample was 1.3, which was 180% higher than that of the pure sample at 773 K. The theoretical efficiency of the Cu_1.8_S + 2 wt.% Fe_3_O_4_ sample was 9.6% (Figure S7, Supporting Information). More critically, the average ZT of 0.57 for the Cu_1.8_S + 2 wt.% Fe_3_O_4_ sample over the entire temperature range was a relatively high value for Cu_1.8_S material systems.

**Figure 5:**
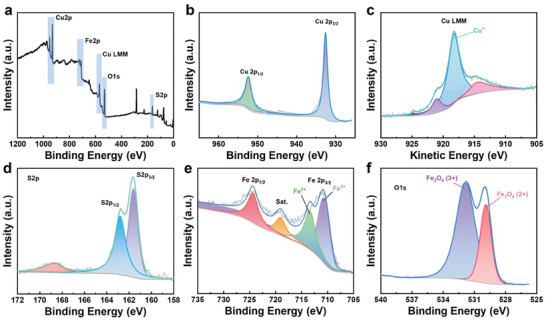
XPS characterization of the Cu_1.8_S + 2 wt.% Fe_3_O_4_ sample. a) XPS survey scan. The observed peaks include b) Cu 2p, c) Cu Lmm Auger, d) S 2p, e) Fe 2p, and (f) O 1s.

To investigate the valence states of the Cu_1.8_S + 2 wt.% Fe_3_O_4_ sample, XPS was performed, and the results are shown in **Figure**  **5**. The full survey scanning results (Figure  5a) demonstrate the existence of Cu 2p, Fe 2p, Cu Lmm, O 1s, and S 2p. Figure  5b shows the Cu 2p spectrum containing two binding energy peaks, Cu 2p_1/2_ and 2p_3/2_. Because the positions of the binding energies of Cu are similar, visually distinguishing the valence of Cu is usually difficult. The characteristic of monovalent Cu is that it exhibits a small satellite peak. Metal Cu showed no satellite peak, whereas divalent Cu showed a strong satellite peak. To thoroughly determine the valence state of Cu, the Auger spectrum peak was examined, and the results are shown in Figure  5c. The results indicate that Cu was monovalent in the Cu_1.8_S + 2 wt.% Fe_3_O_4_ sample. Figure  5d shows the binding energy peaks of S 2p_1/2_ and S 2p_3/2_ at 162.8 and 161.6 eV, respectively. In addition, Fe 2p_1/2_, satellite, and 2p_3/2_ peaks were detected, demonstrating the coexistence of divalent and trivalent iron (Figure  5e). Furthermore, an O 1s peak was detected (Figure  5f). The results show the coexistence of Fe^2+^ and Fe^3+^. These results confirmed the existence of Fe_3_O_4_ and Cu‐rich phases (Cu_1.96_S).

### 2.3 TEM Analysis

**Figure 6:**
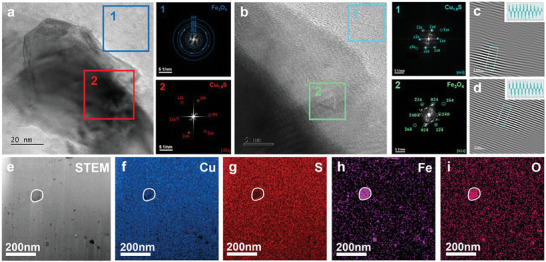
TEM characterization of the Cu_1.8_S + 2 wt.% Fe_3_O_4_ bulk sample. a) HAADF images of the three different contrast regions and corresponding FFT images. b) HRTEM image in (a) indicating the second‐phase region around the interface; c) IFFT image of region 1; and d) IFFT image of region 2. e) Low‐magnification HAADF‐STEM image. EDS mapping of f) Cu, g) S, h) Fe, and i) O, indicating that the precipitates were mainly iron oxides.

To verify the existing defects at the nanoscale, the Cu_1.8_S + 2 wt.% Fe_3_O_4_ sample was analyzed by transmission electron microscopy (TEM). Enlarged high‐angle annular dark‐field (HAADF) images are shown in **Figure**  **6**a. Different contrast regions were observed in the images, with the inset corresponding to fast Fourier transform (FFT) images. The calibration results of the diffraction spots indicate that the second phase was Fe_3_O_4_, which is consistent with the EDS mapping results. Figure  6b shows the high‐resolution TEM (HRTEM) images of the interface region and the corresponding FFT images. The results demonstrate that Fe_3_O_4_ was embedded in the matrix (the triangular precipitated phase in region 2). The inverse fast Fourier transform (IFFT) of regions 1 (Cu_1.8_S) and 2 (Fe_3_O_4_) are shown in Figure  6c,d, respectively. As can be observed, no high‐density dislocations exist. The lattice spacing was measured from IFFT images. Regions 1 and 2 had lattice spacings of 2.099 and 1.813 Å, respectively. Figure  6e shows the HAADF‐STEM images of the Cu_1.8_S + 2 wt.% Fe_3_O_4_ sample. The different contrast regions were due to composition variations. The elemental distribution of iron and oxygen was relatively obvious, and copper and sulfur were uniformly distributed in the matrix. In addition, the Cu‐rich phase (Cu_1.96_S) was also observed in the matrix, as shown in Figure S1 (Supporting Information).

**Figure 7:**
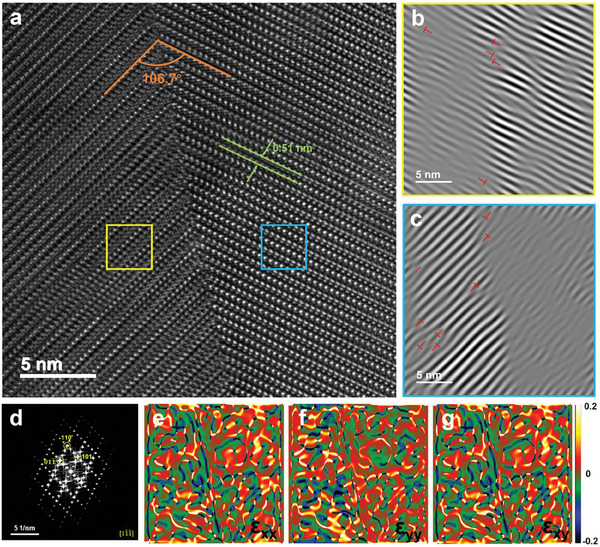
Atomic‐resolution HAADF TEM image of the phase interface region. a) Twinning‐like boundary in the matrix area, b) IFFT image of the yellow rectangular box region in (a), c) IFFT image of the blue rectangular box region in (a), and d) corresponding FFT image. The stress distribution of the entire region in a) was analyzed using GPA, and the color bar represents −20%–20% strain.

**Figure**  **7**a shows that the Cu_1.8_S + 2 wt.% Fe_3_O_4_ sample exhibited a twinning‐like boundary in the matrix, which was confirmed by the FFT patterns presented in Figure  7d. In this study, the cause of the twinning‐like boundary is mainly due to the lattice strain caused by the second phase. For example, the influence of stress or defects in the process of crystal growth leads to local dislocation of the arrangement of atoms, thus forming a twinning‐like boundary. The stress analysis of the entire twinning‐like boundary region (Figure  7e–g) also proves that the twinning‐like boundary has two different stress distributions. The checkerboard‐like diffraction spots correspond to the Na structure in the Cu─S system. The marked spots (yellow circles) are coherent with the hexagonal phase. Through IFFT, we observed that dislocations existed, as depicted in Figure  7b,c. The geometric phase analysis (GPA) results are shown in Figure  7e–g, and the stress distribution around the twin boundary can also be observed. Local concentrated stress existed around the dislocations, which effectively scattered the mid‐frequency phonons and decreased the lattice thermal conductivity over the entire temperature range.^[^ ^22^ ^]^ The structural evolution caused lattice stress due to the formation of additional nanoprecipitates and grain refinement, which introduced numerous interfaces. This resulted in grain extrusion and dislocations distributed at the interfaces. In addition, the existence of dislocations effectively enhances the mechanical properties of reinforced composites. The results are detailed below.

### 2.4 Magnetic Properties

**Figure 8:**
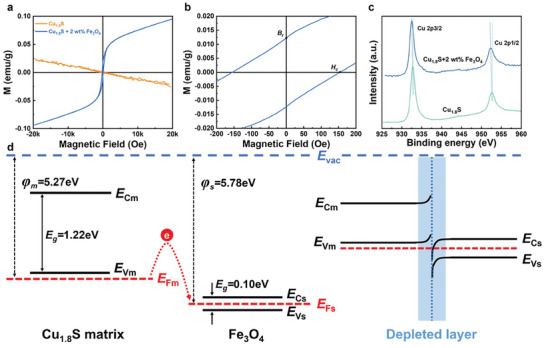
Measured magnetic properties of the specimens and the interface diagram between Cu_1.8_S and Fe_3_O_4_. a) Magnetization vs. magnetic field (M–H) plots of the pure and Cu_1.8_S +2 wt.% Fe_3_O_4_ samples at room temperature. b) M–H plots near the zero field (H = 0). c) The chemical shift toward lower binding energies via XPS demonstrates the occurrence of charge transfer. d) Electron structure of the Cu_1.8_S–Fe_3_O_4_ interface and schematic diagram of the interface barrier between Cu_1.8_S matrix and Fe_3_O_4_ particles.

The magnetic properties and interface diagrams of the pure and Cu_1.8_S + 2 wt.% Fe_3_O_4_ samples are shown in **Figure**  **8**. Br and Hc represent the remnant magnetization and coercive force, respectively. The M─H curves of the Cu_1.8_S + 2 wt.% Fe_3_O_4_ sample demonstrated that the M value increased with H and reached saturation when H reached a certain level. As a typical soft magnetic material, the addition of Fe_3_O_4_ triggered the magnetic transformation of Cu_1.8_S. Cu_1.8_S exhibited weak magnetism owing to the presence of Fe_3_O_4_. The previous study showed that the scattering parameter of the matrix (ϒ0) is beneficial for the Seebeck coefficient (S) but does not affect the effective mass. The relationship between ϒ0 and S was calculated using the following equation: $S=\left[\frac{8\pi^{2}k_{B}^{2}}{3eh^{2}}\right]m^{*}T{\left[\frac{\pi }{3n}\right]}^{\frac{2}{3}}\left(\Upsilon +1\right)$.^[^ ^23^ ^]^ The work function of magnetic Fe_3_O_4_ (φ = 5.5 eV)^[^ ^24^ ^]^ was larger than that of the Cu_1.8_S matrix (φ = 5.27 eV),^[^ ^25^ ^]^ which may have created barriers for hole carriers to reduce the pure sample's high carrier concentration (≈5.56 × 1020 cm^−3^) to the ideal interval (≈2.5 × 1020 cm^−3^).^[^ ^26^ ^]^ The difference in work function between Cu_1.8_S and Fe_3_O_4_, thus a depleted layer would be generated near the interface when the carrier transports. This potential barrier would scatter low‐energy carriers while allowing high‐energy transport, resulting in the slightly improved Seebeck coefficient of the samples. Through X‐ray photoelectron spectroscopy (XPS) characterization, we observed a chemical shift from a high binding energy to a low binding energy, and the results demonstrate that charge transfer occurred.^[^ ^23^, ^27^ ^]^ As depicted in the diagram, the charge transfer forms an interface potential barrier. In this study, the Seebeck coefficient after adding Fe_3_O_4_ at RT was consistent with the above trend, indicating that iron oxide is beneficial to the Seebeck coefficient of copper sulfides. These results demonstrate that adding a promoted number of magnetic Fe_3_O_4_ particles to the matrix not only introduces extra electrons and decreases the carrier concentration but also enhances the scattering of electrons. Therefore, as the Fe_3_O_4_ content increased, the carrier concentration and mobility gradually decreased, as shown in Figure S7 (Supporting Information).

### 2.5 Mechanical Properties

**Figure 9:**
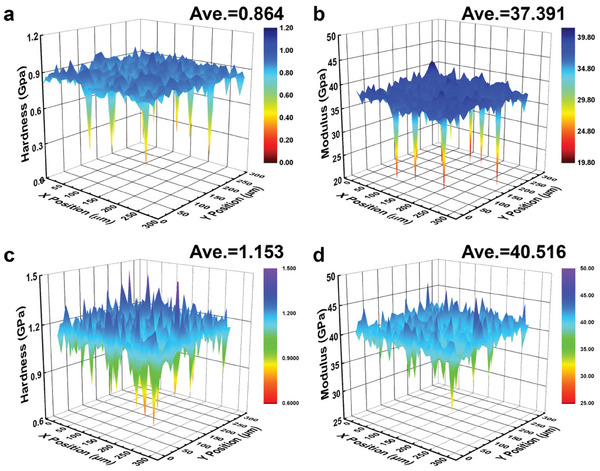
Mechanical performance characterization using a nanoindentation instrument. 3D cloud diagrams showing the a) hardness and b) modulus of the Cu_1.8_S pure sample. 3D cloud diagrams showing the c) hardness and d) modulus of the Cu_1.8_S + 2 wt.% Fe_3_O_4_ sample.

The mechanical performances of the pure and Cu_1.8_S + 2 wt.% Fe_3_O_4_ samples were characterized via nanoindentation using the Nano‐Blitz 3D method (**Figure**  **9**). The average hardness of the pure sample was 0.864 GPa, which was lower than the hardness of the Cu_1.8_S + 2 wt.% Fe_3_O_4_ sample. After adding Fe_3_O_4_, the modulus of the sample also improved. The overall mechanical properties of the Cu_1.8_S+2 wt.% Fe_3_O_4_ sample were increased by 35%. The mechanical performance was optimized by second‐phase strengthening. A large number of interfaces and phases were introduced via mechanical alloying, which effectively hindered dislocation movement and thus enhanced the mechanical properties. The improved mechanical performance contributes to the work stability of p‐type legs in copper sulfide TE devices.

## 3 Conclusion

In this study, we propose a strategy for constructing copper sulfide magnetic nanocomposites by drawing lessons from the smelting process of Cu minerals. In situ, solid‐state chemical reactions achieve the modulation of carrier concentration, followed by the composition and microstructure control. The ferromagnetic Fe_3_O_4_ facilitated charge transfer, and engineered the interface barrier potential, effectively enhancing the Seebeck coefficient. Benefiting from the enhanced Seebeck coefficient, the average power factor was increased by 26% over the entire operating temperature range. In addition, phase boundaries, dislocations, and precipitates effectively suppress the thermal conductivity of porous magnetic nanocomposites over the entire temperature range. The thermal conductivity of the doped sample was 70% lower than that of the pure sample. The mechanical properties were effectively enhanced by 35%, as characterized by nanoindentation. Based on the optimization of the power factor and thermal conductivity over the entire temperature range, the ZT value of the Cu_1.8_S + 2 wt.% Fe_3_O_4_ sample was 1.3 at 773 K, which was 180% higher than that of the pure sample. The average ZT was 0.57 in the temperature range of 323–773 K, which is the highest average ZT value of the Cu─S material system. This work demonstrated that the use of oxides is a high‐efficiency strategy to regulate the carrier concentration of metal sulfides, and this strategy can also be used in other fields of sulfide functional materials.

## Conflict of Interest

The authors declare no conflict of interest.

## Supporting information



Supporting Information

## Data Availability

The data that support the findings of this study are available from the corresponding author upon reasonable request.

## References

[1] a) S. Ayachi , X. He , H. J. Yoon , Adv. Energy Mater. 2023, 13, 2300937;

[2] a) D. M. Rove , in Thermoelectrics Handbook, (Ed: D. W. Rowe ), Boca Raton, Florida 2006, 1, pp. 1–10;

[3] a) H. Liu , X. Shi , F. Xu , L. Zhang , W. Zhang , L. Chen , Q. Li , C. Uher , T. Day , G. J. Snyder , Nat. Mater. 2012, 11, 422; 22406814 10.1038/nmat3273

[4] a) W. Zhou , H. Z. Li , Z. H. Shan , R. Zhang , S. K. Lu , J. Pei , Z. H. Ge , M. Zhou , Y. B. Wang , B. P. Zhang , Sci. China Mater. 2023, 66, 2051;

[5] a) S. Zhao , H. Chen , X. Zhao , J. Luo , Z. Tang , G. Zeng , K. Yang , Z. Wei , W. Wen , X. Chen , Y. Sun , Mater. Today Phys. 2020, 15, 100271;

[6] a) B. B. Jiang , W. Wang , S. X. Liu , Y. Wang , C. F. Wang , Y. N. Chen , L. Xie , M. Y. Huang , J. Q. He , Science 2022, 377, 208; 35857539 10.1126/science.abq5815

[7] a) H. C. Tang , F. H. Sun , J. F. Dong , Asfandiyar, H. L. Z , Y. Pan , J. F. Li , Nano Energy 2018, 49, 267;

[8] a) T. Mao , P. Qiu , P. Hu , X. Du , K. Zhao , T. R. Wei , J. Xiao , X. Shi , L. Chen , Adv. Sci. (Weinh) 2020, 7, 1901598; 31921552 10.1002/advs.201901598 PMC6947709

[9] a) Y. Pei , X. Shi , A. LaLonde , H. Wang , L. Chen , G. J. Snyder , Nature 2011, 473, 66; 21544143 10.1038/nature09996

[10] a) L. Hu , Y. B. Luo , Y. W. Fang , F. Y. Qin , X. Cao , H. Y. Xie , J. W. Liu , J. F. Dong , A. Sanson , M. Giarola , X. Y. Tan , Y. Zheng , A. Suwardi , Y. Z. Huang , K. Hippalgaonkar , J. Q. He , W. Q. Zhang , J. W. Xu , Q. Y. Yan , M. G. Kanatzidis , Adv. Energy Mater. 2021, 11, 2100661;

[11] a) Y. Qin , B. Qin , T. Hong , X. Zhang , D. Wang , D. Liu , Z. Y. Wang , L. Su , S. Wang , X. Gao , Z. H. Ge , L. D. Zhao , Science 2024, 383, 1204; 38484057 10.1126/science.adk9589

[12] a) Y. He , P. Lu , X. Shi , F. Xu , T. Zhang , G. J. Snyder , C. Uher , L. Chen , Adv. Mater. 2015, 27, 3639; 25962487 10.1002/adma.201501030

[13] Z. Zulhan , I. M. Fauzian , T. Hidayat , J. Mater. Res. Technol. 2020, 9, 13625.

[14] a) I. Wilkomirsky , R. Parra , F. Parada , E. Balladares , E. Seguel , J. Etcheverry , R. Díaz , Metall. Mater. Trans. B 2020, 51, 1540.

[15] W. Jin , S. Yang , C. Tang , Y. Li , C. Chang , Y. Chen , ACS Sustainable Chem. Eng. 2023, 11, 9932.

[16] a) Y. Yao , B.‐P. Zhang , J. Pei , Q. Sun , G. Nie , W.‐Z. Zhang , Z.‐T. Zhuo , W. Zhou , ACS Appl. Mater. Interfaces 2018, 10, 32201; 30178653 10.1021/acsami.8b11300

[17] a) S. W. Gu , P. Qin , Y. X. Zhang , J. Guo , Q. Shan , J. Feng , Z. H. Ge , J. Alloy. Compd. 2021, 852, 156972;

[18] a) Y. X. Zhang , T. Y. Yang , Z. Y. Wang , J. Feng , Z. H. Ge , Mater. Today Phys. 2022, 27, 100808;

[19] Y. X. Zhang , Z. H. Ge , J. Feng , J. Alloy. Compd. 2017, 727, 1076.

[20] S. Sailler , R. Bueno Villoro , S. Bayesteh , H. Schlörb , M. O. Cichocka , K. Nielsch , S. Zhang , N. Pérez , Mater. Today Phys. 2024, 46, 101477.

[21] Y. Gong , W. Dou , B. Lu , X. Zhang , H. Zhu , P. Ying , Q. Zhang , Y. Liu , Y. Li , X. Huang , M. F. Iqbal , S. Zhang , D. Li , Y. Zhang , H. Wu , G. Tang , Nat. Commun. 2024, 15, 4231. 38762611 10.1038/s41467-024-48635-0 PMC11102544

[22] a) S. I. Kim , K. H. Lee , H. A. Mun , H. S. Kim , S. W. Hwang , J. W. Roh , D. J. Yang , W. H. Shin , X. S. Li , Y. H. Lee , G. J. Snyder , S. W. Kim , Science 2015, 348, 109; 25838382 10.1126/science.aaa4166

[23] a) W. Zhao , Z. Liu , Z. Sun , Q. Zhang , P. Wei , X. Mu , H. Zhou , C. Li , S. Ma , D. He , P. Ji , W. Zhu , X. Nie , X. Su , X. Tang , B. Shen , X. Dong , J. Yang , Y. Liu , J. Shi , Nature 2017, 549, 247; 28905895 10.1038/nature23667

[24] M. Zervos , Z. Viskadourakis , G. Athanasopoulos , R. Flores , O. Conde , J. Giapintzakis , J. Appl. Phys. 2014, 115, 033709.

[25] a) G. Liu , T. Schulmeyer , J. Brötz , A. Klein , W. Jaegermann , Thin Solid Films 2003, 431, 477;

[26] M. Hong , J. Zou , Z. G. Chen , Adv. Mater. 2019, 31, 1807071. 10.1002/adma.201807071 30756468

[27] a) W. Zhao , Z. Liu , P. Wei , Q. Zhang , W. Zhu , X. Su , X. Tang , J. Yang , Y. Liu , J. Shi , Y. Chao , S. Lin , Y. Pei , Nat. Nanotechnol. 2017, 12, 55; 27723733 10.1038/nnano.2016.182

