# Supplementary material for: Excellent Thermoelectric Performance Realized in Copper Sulfide Magnetic Nanocomposites Via Modified Solid States Reaction
Source: Adv Sci (Weinh). 2025 Feb 8;12(13):2409494. doi: 10.1002/advs.202409494 (PMC11967778; doi:10.1002/advs.202409494)
Supplement: Supplementary file 1 — Supporting Information [file ADVS-12-2409494-s001.docx]

Supporting Information

Excellent Thermoelectric Performance Realized in Copper Sulfide Magnetic Nanocomposites via Modified Solid States Reaction

Tian-Yu Yang, Zi-Yuan Wang, Xi Yan, Chong-Yu Wang, Yi-Xin Zhang, Zhen-Hua Ge*, Jing Feng*

Faculty of Materials Science and Engineering, Kunming University of Science and Technology, Kunming 650093, China

E-mail: z.ge@kust.edu.cn, jingfeng@kust.edu.cn

Experimental Section

*Synthesis*: Cu_1.8_S + *x* wt.% Fe_3_O_4_ (*x* = 0, 0.5, 1, 1.5, 2) powder samples in this study were prepared using stoichiometrically weighted Cu (99.99%, <200 mesh), S (99.99%, <200 mesh), and Fe_3_O_4_ powders (99.99%). They were then placed in stainless steel vessels with a ratio of stainless steel balls to materials of 20:1 under protective atmospheric conditions (argon–hydrogen mixture). The MA procedure was performed for 2 h at 425 rpm in a planetary ball milling machine. The mixtures were then placed in a *Φ*20mm graphite mold and sintered at 773 K under 50 MPa for 5 min in an SPS system (Sumitomo SPS1050, Japan). The sintered bulk samples were cut into specific shapes and polished for characterization.

*Characterization*: The phase structures of the samples were characterized by XRD (MiniFlex600 Rigaku, Japan) from 20° to 60° at a scan rate of 10°/min with Cu *K_α_* radiation (*λ* = 1.54 Å). The fracture morphologies of the samples were observed by field-emission scanning electron microscopy (FESEM, ZEISS, Sigma 300, Germany). Scanning TEM (STEM, FEI Titan Cube) was used to observe the microstructures and nanoprecipitates in the bulk samples. The elemental distribution of the samples was characterized by electron probe microanalysis (EPMA, JEOL, JXA-8230, Japan). The electrical and thermal properties of the samples were characterized using a resistivity and Seebeck coefficient measurement system (CTA-3, China) and a laser thermal conductivity meter (NETZSCH LFA-467, Germany), respectively. The specific heat capacity (*C*_p_) was calculated using the Neumann–Kopp rule, and the mass density (*ρ*) was obtained using the Archimedes method. The carrier concentration (*n_H_*) and mobility (*μ_H_*) of the samples were measured at RT using a Hall effect measurement system (Ecopia, HMS-7000, Korea). Vickers hardness and Young modulus were measured using a nanoindenter by pressing 900 grids with a load of 20 mN (iMicro KLA, USA). The absolute errors of the measured properties were estimated to be 3%–4%, and the error bars of ZT were 15%–20%.


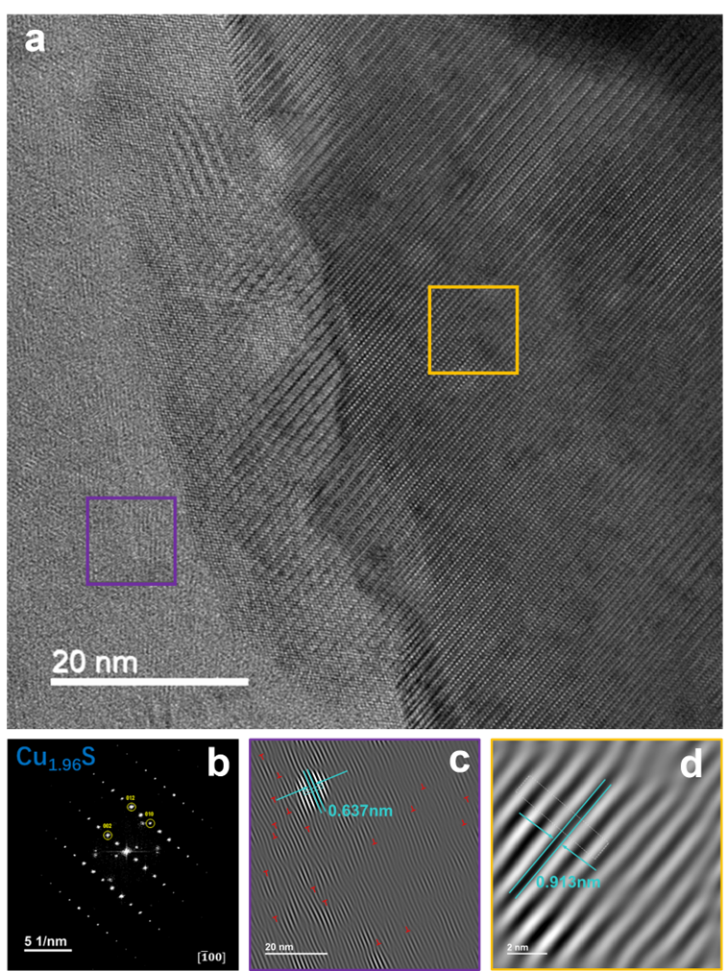


Figure S1. (a) Enlarged HAADF image showing a Cu-rich area, (b) corresponding FFT image of the entire area, (c) IFFT image of the purple rectangular box region in (a), and (d) IFFT image of the orange rectangular box region in (a), indicating that local stress existed in the different contrast regions and uneven dislocations formed in some areas.

In the SPS process, the Fe_3_O_4_ added to the matrix caused SO_2_ gas to emerge, and the matrix generated a Cu-rich phase, namely Cu_1.96_S (Figure. S1a). Because local stress existed, the second-phase region experienced several dislocations. As shown in Figure. S1b, the FFT images are coherent with Cu_1.96_S. These results demonstrate that the existence of a second phase and dislocations can effectively scatter phonons and suppress thermal conductivity.


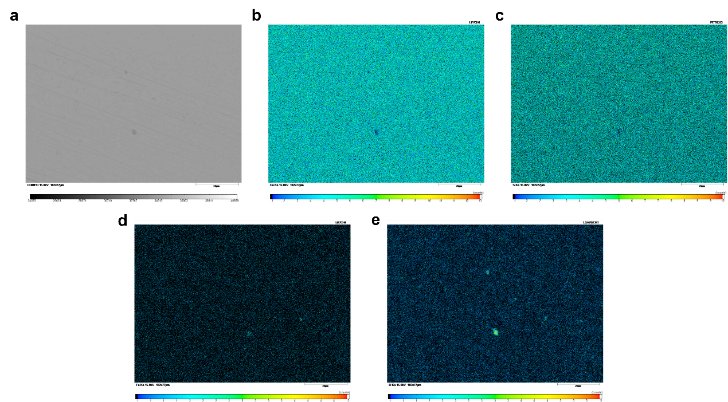


Figure S2. EPMA mapping of the 2 wt% Fe_3_O_4_-doped sample: (a) BSE images and elemental maps of (b) Cu, (c) S, (d) Fe, and (e) O.


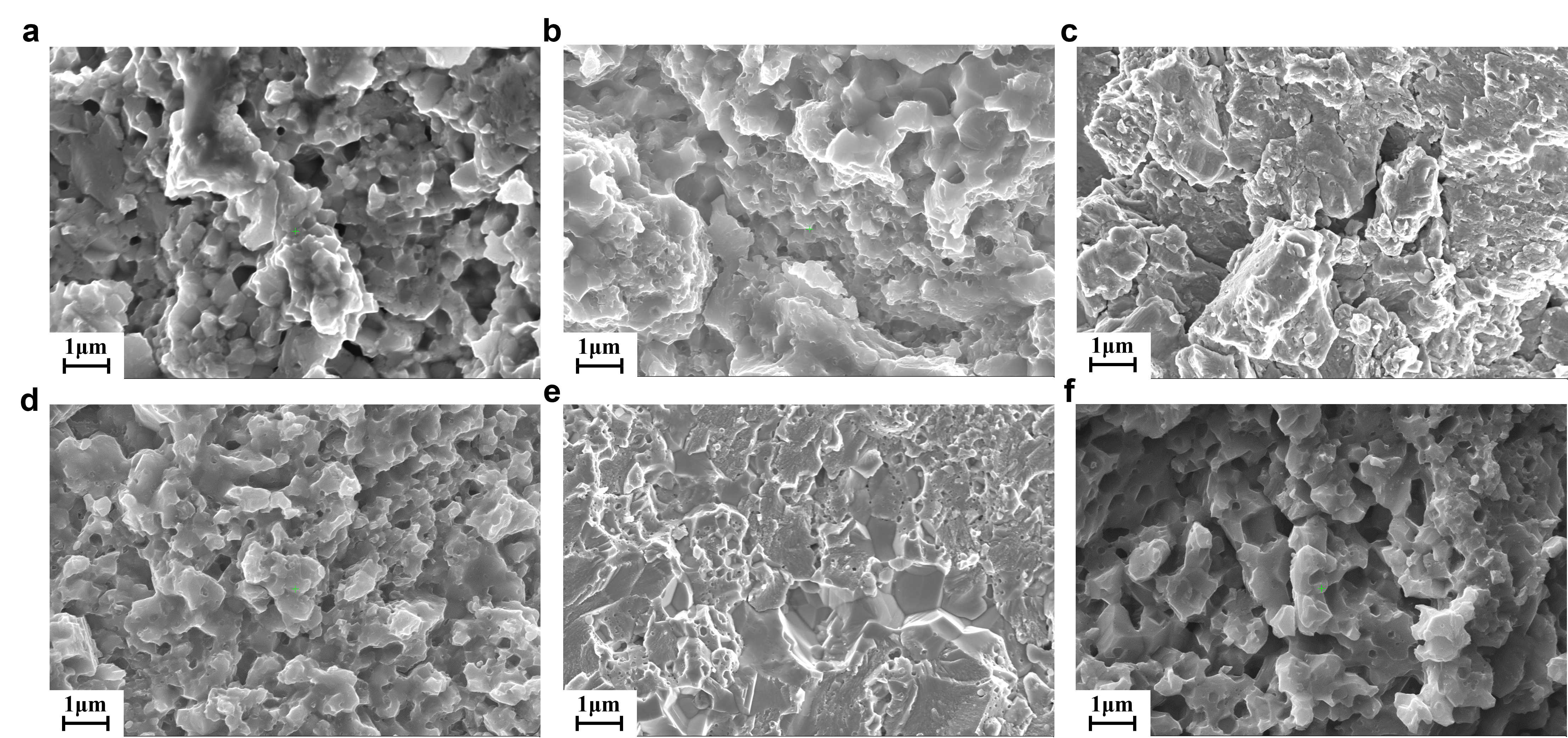


Figure S3. Fracture morphologies of the Cu_1.8_S + *x* wt% Fe_3_O_4_ (*x* = 0, 0.5, 1, 1.5, 2, 2.5) bulk samples: (a) pure, (b) *x* = 0.5, (c) *x* = 1, (d) *x* = 1.5, (e) *x* = 2, and (f) *x* = 2.5.

The fracture morphologies of the Cu_1.8_S + *x* wt% Fe_3_O_4_ (*x* = 0, 0.5, 1, 1.5, 2, 2.5) bulk samples were characterized by scanning electron microscopy (SEM) (Figure. S3). The sample density is presented in Figure. S5. Nanopores were introduced after the addition of Fe_3_O_4_, and pristine samples existed in some pores because of sulfur volatilization during the SPS process. Previous studies have demonstrated that the structure evolution of copper sulfides is caused by compositional differences and that the tiny pores distributed in grain boundaries are due to the different thermal expansions between the Cu_1.8_S matrix and nanoprecipitates.^[1]^


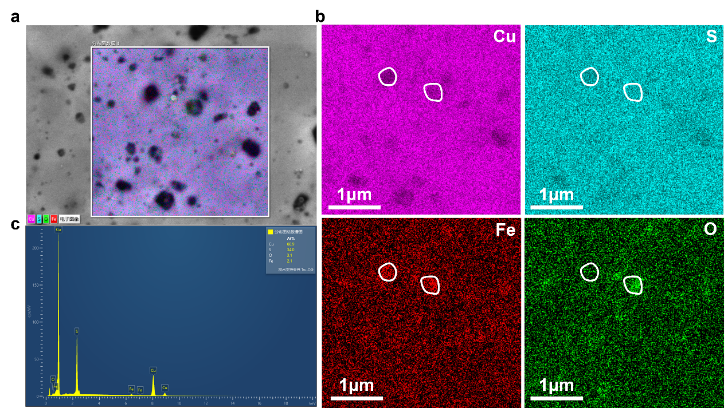


Figure S4. EDS mapping of the Cu_1.8_S + 2 wt% Fe_3_O_4_ sample. (a) SEM image, (b) element mapping image, and (c) elemental distribution spectrum.

The energy-dispersive spectroscopy (EDS) mapping results were obtained in different regions (Figure. S4). We observed the existence of Fe_3_O_4_. In addition, the diffusion of oxygen was observed, which is inferred to be caused by the decomposition of Fe_3_O_4_ and incomplete reaction. These results corresponded to the previous conclusion—that is, the special nanoprecipitates are usually embedded in nanopores in copper chalcogenides, and this phenomenon is due to element emission and the existence of a suitable substrate for crystal growth.


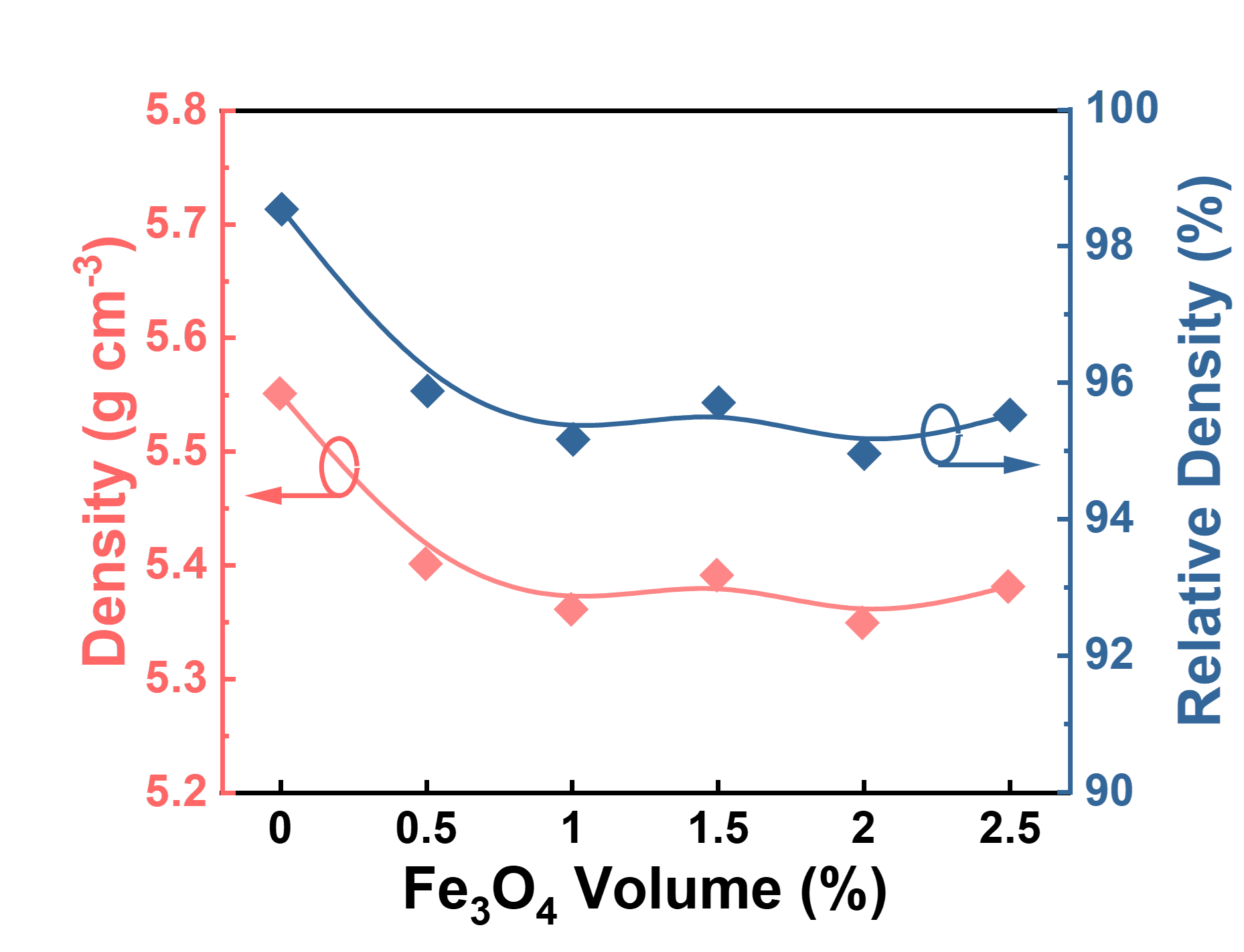


Figure S5. Density and relative density of the Fe_3_O_4_-doped samples.


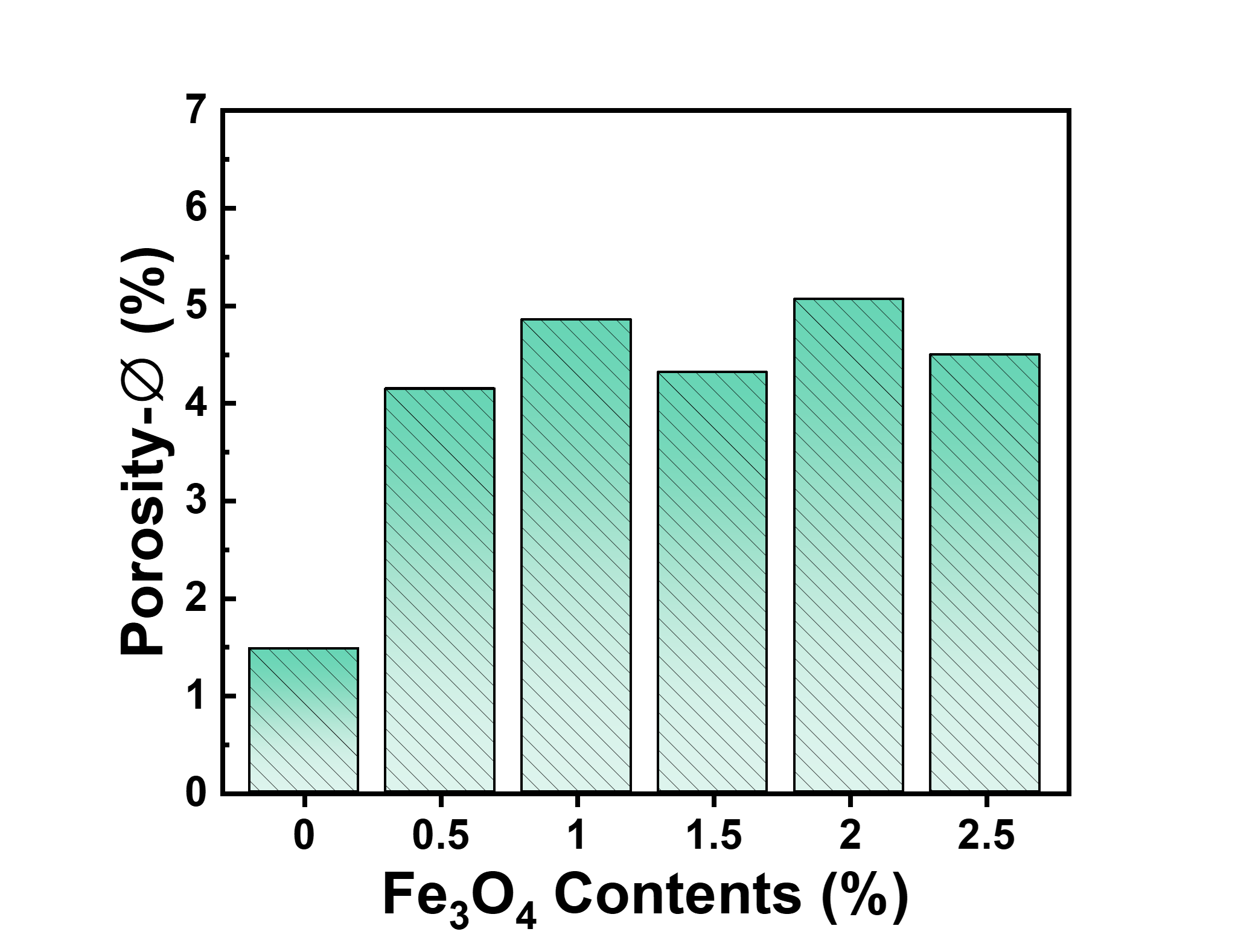


Figure S6. Porosity of the Fe_3_O_4_-doped samples.


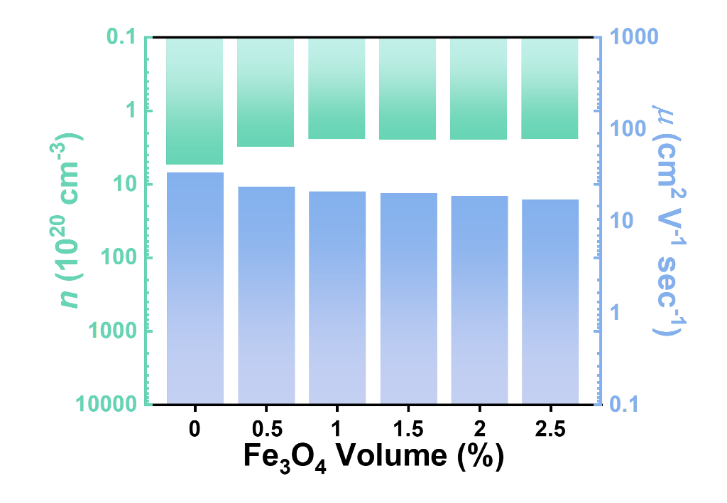


Figure S7. Carrier concentration and mobility of the samples.


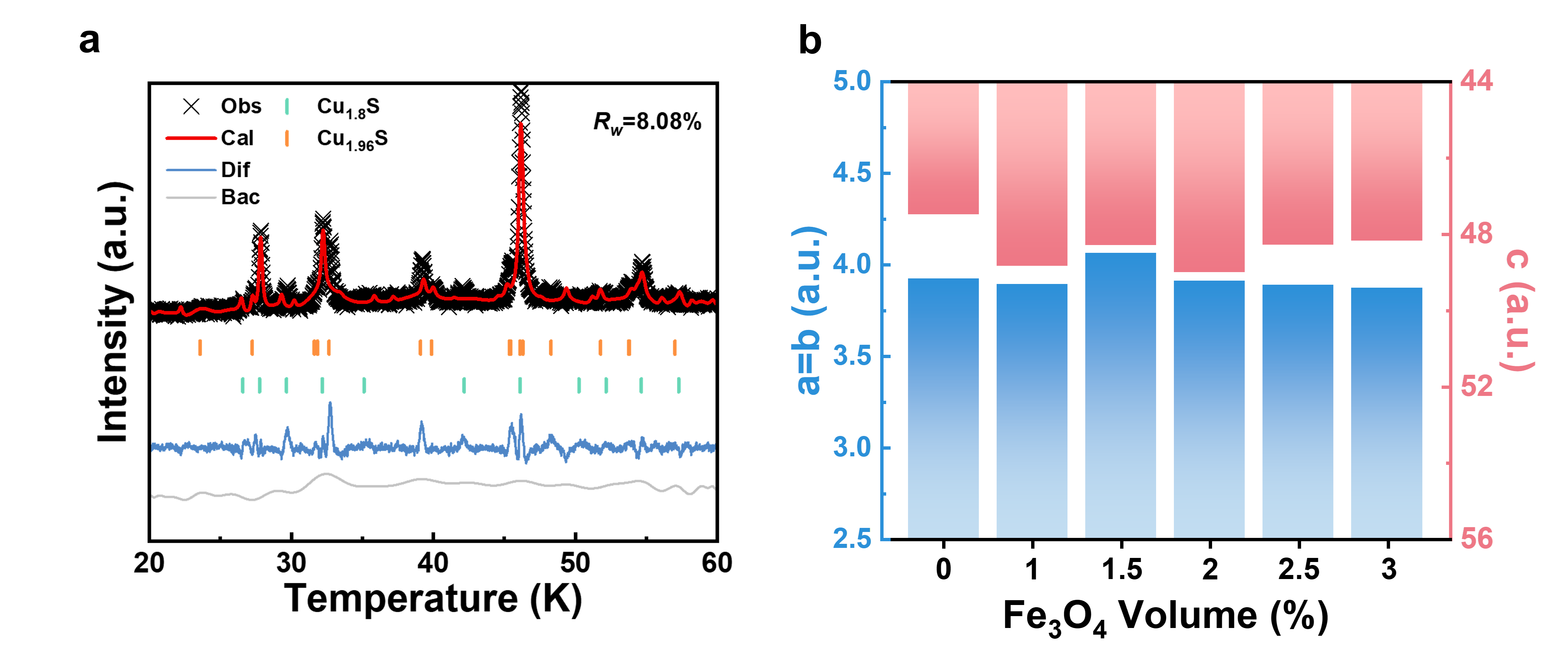


Figure S8. (a) XRD Rietveld refinement data and (b) lattice constants of the samples.


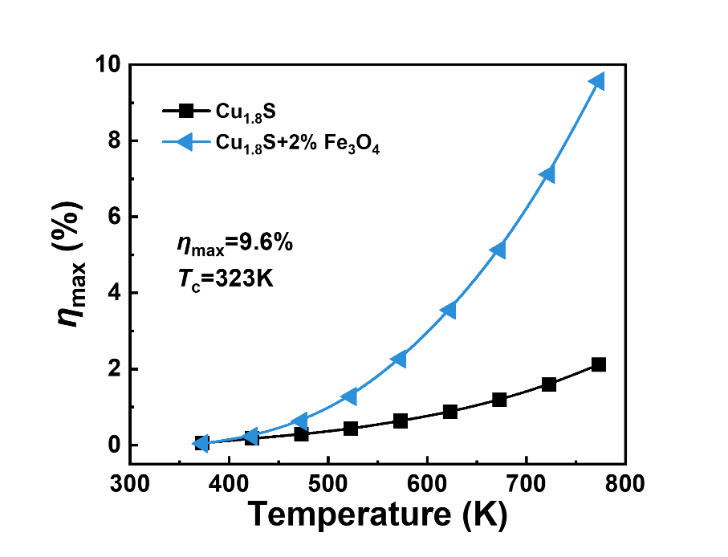


Figure S9. Theoretical efficiency of 2 wt% Fe_3_O_4_ doped sample.


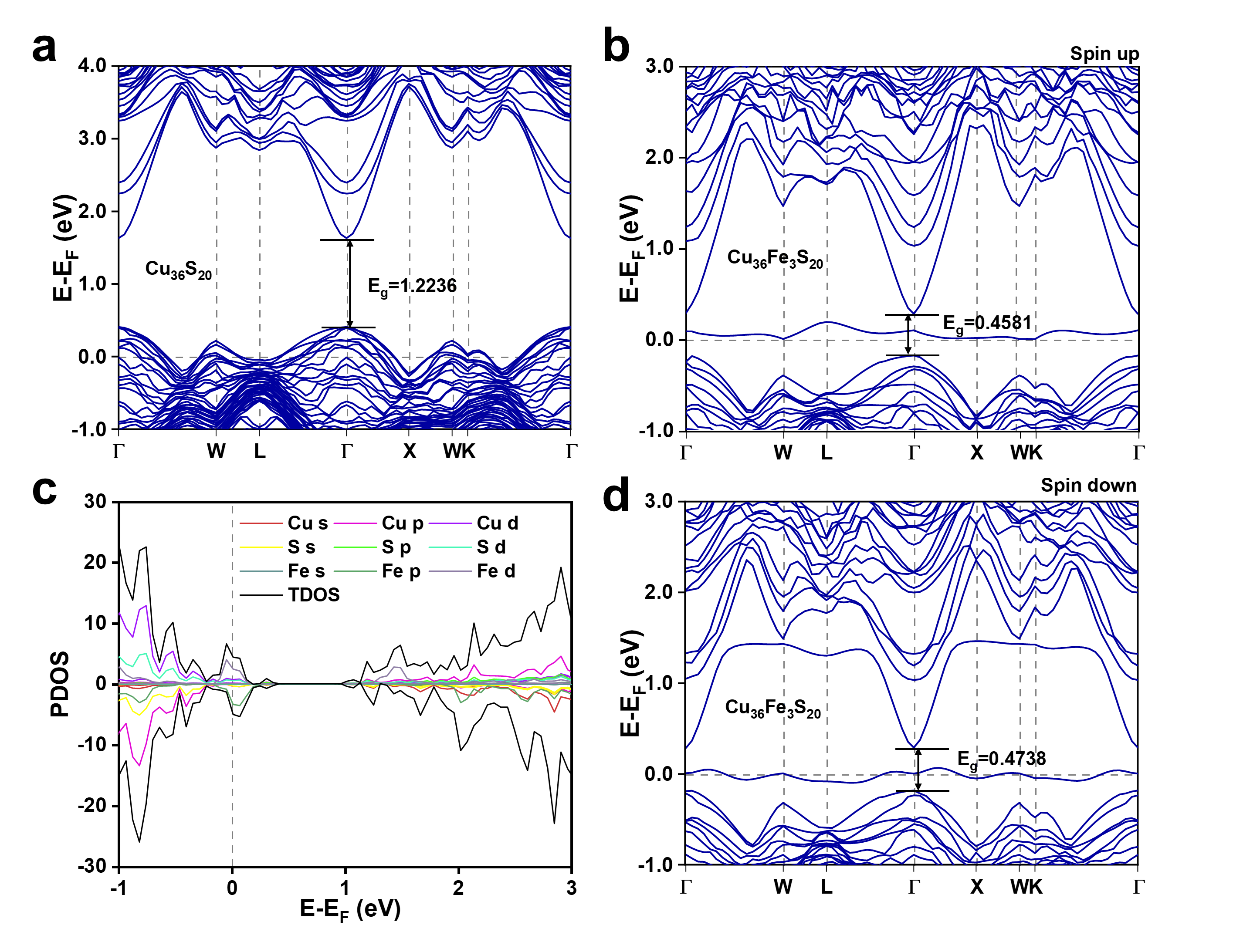


Figure S10. The electronic band structure of a) Cu_36_S_20_ and b-d) Cu_36_Fe_3_S_20_. c) The projected density of states of doped sample.

The influence of the introduction of ferromagnetic particles on the electrical properties of the materials is as follows. First, the change of the electronic band structure. As shown in Figure S10, the band structure changes after the addition of magnetic elements. Compared with the position of the Fermi level in Cu_1.8_S, the Fermi level moves up and the hole concentration decreases after the addition of Fe element, which corresponding to the data of Figure S7. Besides, according to the calculation of the density of states, as shown in Figure S10 c, the addition of ferromagnetic particles effectively improves the density of states. The calculation formula of the Seececk coefficient is as follows. ^[2]^

$$S=\frac{\pi^{2}}{3}\frac{k_{B}}{q}k_{B}T{\{\frac{d[ln(\sigma(E))]}{dE}\}}_{E=E_{F}}$$

$$=\frac{\pi^{2}}{3}\frac{k_{B}}{q}k_{B}T{\{\frac{1}{n}\frac{dn\left( E \right)}{dE}+\frac{1}{\mu}\frac{d\mu(E)}{dE}\}}_{E=E_{F}}$$

Equation shows that the density of states $DOS(E)=\frac{dn(E)}{dE}$. According to the formula, the increased of the density of states corresponding to the increase of the Seebeck coefficient, which is also consist with the experimental results. In addition, due to the introduction of foreign magnetic particles, as a new carrier scattering center, some low-energy carriers will be scattered, which reduces the carrier mobility (Figure S7). Finally, beneficial for the optimized Seebeck coefficient, the average power factor of the material has been improved (Figure 1b), which has played a certain role in optimizing the electrical properties.


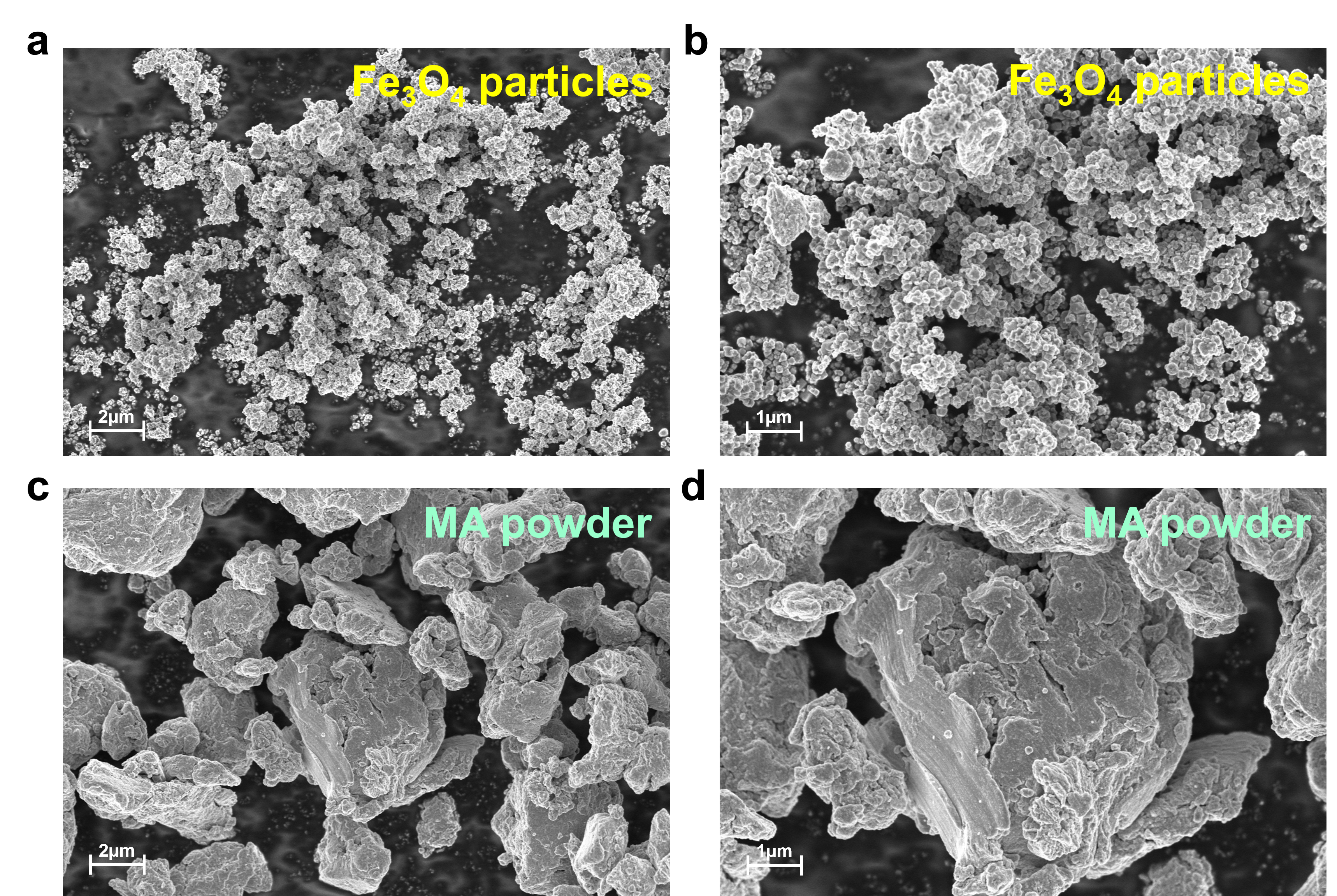


Figure S11. The SEM images of a-b) Fe_3_O_4_ particles and c-d) ball milling powder.

As shown in Figure S11, the Fe_3_O_4_ particles are tiny and dispersed. At a magnification of 10,000 times (Figure S11b), the grain size of Fe_3_O_4_ particles is observed to be about nanometer level and then agglomerated together. The ball milling powder SEM images of copper sulfide adding Fe_3_O_4_ are shown in Figure S11c-d. It can be seen that the grain size of copper sulfide is much larger than that of Fe_3_O_4_ particles at the same magnification. The pores on the surface of the grains are due to the generated SO_2_, and some small second phases with different contrast can be seen on the surface of the grains.


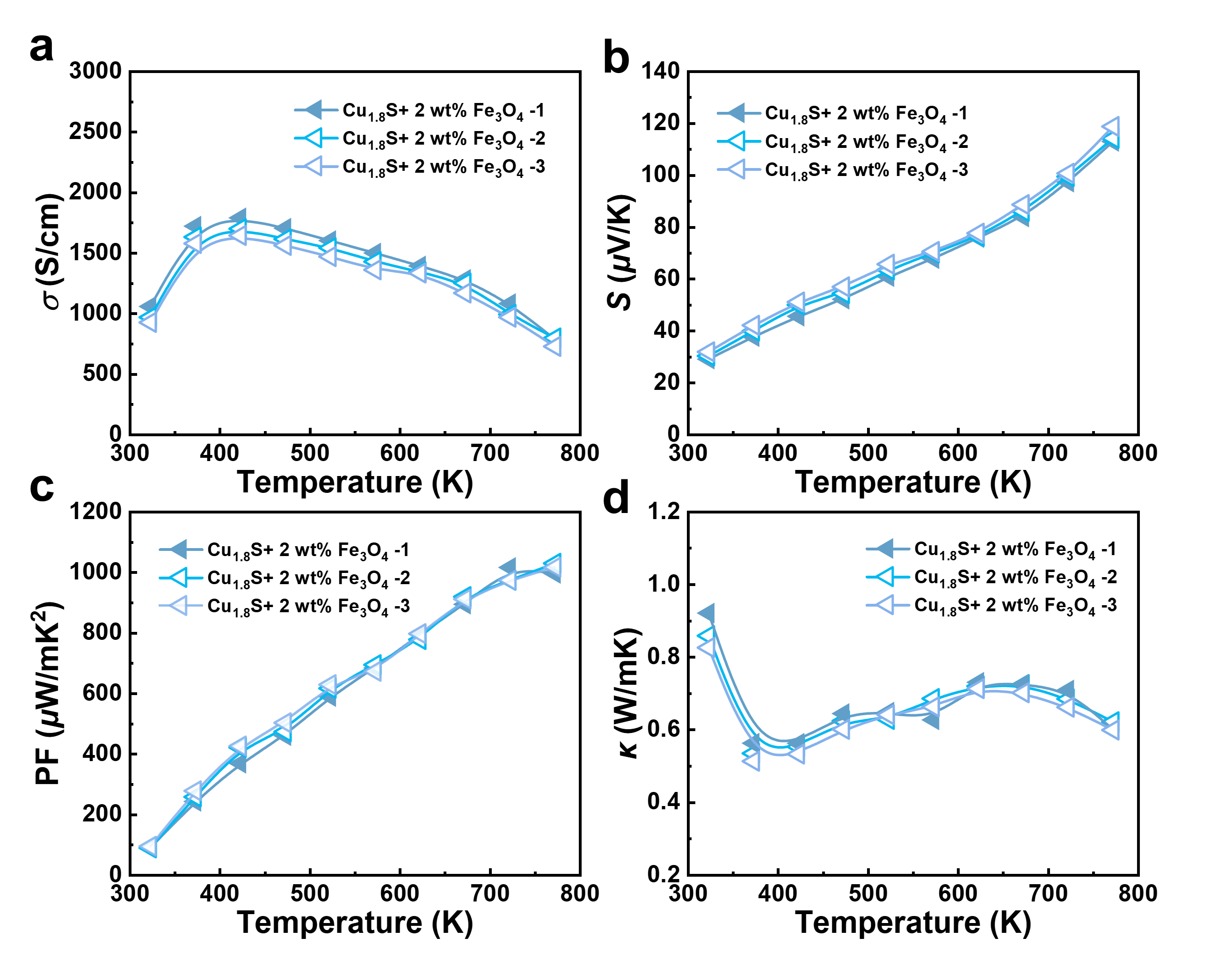


Figure S12. Temperature dependence of a) electrical conductivity, b) Seebeck coefficient, c) power factor, and d) thermal conductivity of the Cu_1.8_S + 2 wt% Fe_3_O_4_ sample in the cycling measurement.


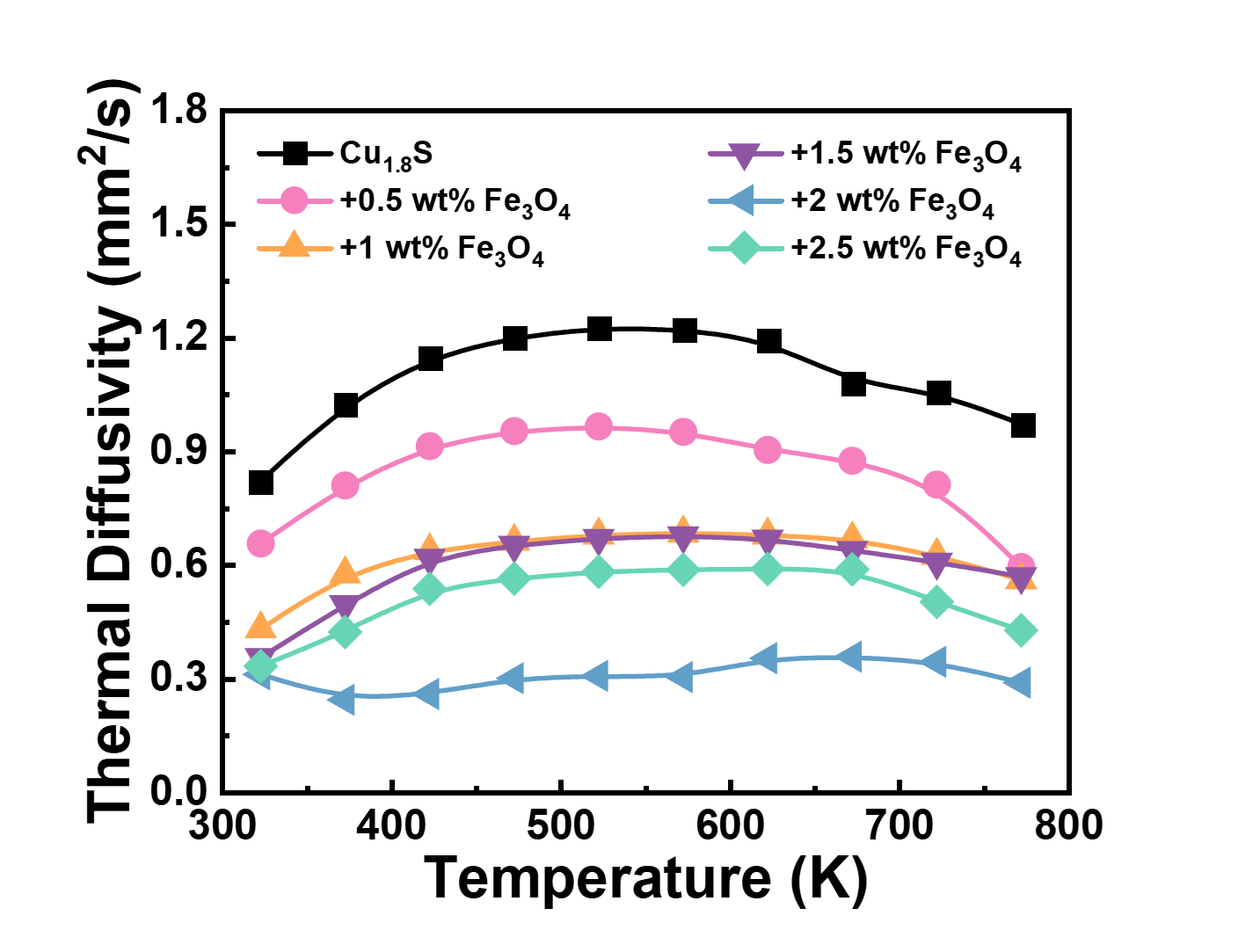


Figure S13. Temperature dependence of thermal diffusivity for Cu_1.8_S + *x* wt% Fe_3_O_4_ (*x* = 0, 0.5, 1, 1.5, 2, 2.5) bulk samples.

Table S1. Density (*ρ*), the sound velocity (*v*_t_, *v*_l_, *v*_a_), Young’s modulus (*E*), shear modulus (*G*), Poisson’s ratio (*r*) and Grüneisen parameters (*γ*) of all Cu_1.8_S + *x* wt% (*x* = 0, 0.5, 1, 1.5, 2, 2.5) Fe_3_O_4_ samples.

| Sample | *v*_t_  (m s^-1^) | *v*_l_  (m s^-1^) | *v*_a_  (m s^-1^) | *E*  (GPa) | *G*  (GPa) | *r* | *γ* |
| --- | --- | --- | --- | --- | --- | --- | --- |
| Cu_1.8_S | 1937.30 | 3657.14 | 2165 | 53.89 | 20.65 | 0.305 | 1.80 |
| 0.5% Fe_3_O_4_ | 1370.48 | 4172.15 | 1560 | 28.93 | 10.05 | 0.439 | 3.17 |
| 1% Fe_3_O_4_ | 1695.47 | 4082.76 | 1918 | 43.59 | 15.61 | 0.396 | 2.58 |
| 1.5% Fe_3_O_4_ | 1701.15 | 3746.84 | 1918 | 42.57 | 15.53 | 0.370 | 2.31 |
| 2% Fe_3_O_4_ | 1723.08 | 4083.98 | 1948 | 44.44 | 15.97 | 0.391 | 2.53 |
| 2.5% Fe_3_O_4_ | 1857.75 | 4527.41 | 2102 | 52.06 | 18.61 | 0.398 | 2.61 |

Average sound velocity (*v*_a_) is calculated from as follows.

$$\frac{1}{v_{a}}={[1/3(\frac{1}{v_{l}^{3}}+\frac{2}{v_{t}^{3}})]}^{1/3}$$

Young’s modulus (*E*) is calculated by

$$E= \frac{\rho v_{t}^{2}(3v_{l}^{2}-4v_{t}^{2})}{(v_{l}^{2}-v_{t}^{2})}$$

Shear modulus (*G*) is calculated by

$$G=\frac{E}{2(1+r)}$$

Poisson’s ratio (*r*) is calculated by

$$r=\frac{1-2{(v_{t}/v_{l})}^{2}}{2-2{(v_{t}/v_{l})}^{2}}$$

The Grüneisen parameter (*γ*) is calculated by

$$\gamma=\frac{3}{2}(\frac{1+r}{2-3r})$$

Reference

[1] Y. X. Zhang, Q. Y. Huang, X. Yan, C. Y. Wang, T. Y. Yang, Z. Y. Wang, Y. C. Shi, Q. Shan, J. Feng, Z. H. Ge, *Nat. Commun.* **2024**, *15*, 2736.

[2] a) Joseph P. Heremans, Vladimir Jovovic, Eric S. Toberer, Ali Saramat, Ken Kurosaki, Anek Charoenphakdee, Shinsuke Yamanaka, G. J. Snyder, *Science* **2008**, 321, 554; b) J. He, T. M. Tritt, *Science* **2017**, 357, 1369.
